# Metabolic Profiling Indicates Diversity in the Metabolic Physiologies Associated With Maternal Postpartum Depressive Symptoms

**DOI:** 10.3389/fpsyt.2021.685656

**Published:** 2021-06-25

**Authors:** Emma Bränn, Christina Malavaki, Emma Fransson, Maria-Konstantina Ioannidi, Hanna E. Henriksson, Fotios C. Papadopoulos, George P. Chrousos, Maria I. Klapa, Alkistis Skalkidou

**Affiliations:** ^1^Department of Women's and Children's Health, Uppsala University, Uppsala, Sweden; ^2^Metabolic Engineering and Systems Biology Laboratory, Institute of Chemical Engineering Sciences, Foundation for Research and Technology-Hellas, Patras, Greece; ^3^Department of Microbiology, Tumor and Cell Biology, Centre for Translational Microbiome Research, Karolinska Institute, Stockholm, Sweden; ^4^Department of Biology, University of Patras, Patras, Greece; ^5^Department of Neuroscience, Psychiatry, Uppsala University, Uppsala, Sweden; ^6^University Research Institute of Maternal and Child Health and Precision Medicine, UNESCO Chair on Adolescent Health Care, Medical School, National and Kapodistrian University of Athens, Athens, Greece

**Keywords:** postpartum depression, metabolomics, perinatal depression, pregnancy, GC-MS metabolic profiling, molecular psychiatry, precision medicine

## Abstract

**Background:** Postpartum depression (PPD) is a devastating disease requiring improvements in diagnosis and prevention. Blood metabolomics identifies biological markers discriminatory between women with and those without antenatal depressive symptoms. Whether this cutting-edge method can be applied to postpartum depressive symptoms merits further investigation.

**Methods:** As a substudy within the Biology, Affect, Stress, Imagine and Cognition Study, 24 women with PPD symptom (PPDS) assessment at 6 weeks postpartum were included. Controls were selected as having a score of ≤ 6 and PPDS cases as ≥12 on the Edinburgh Postnatal Depression Scale. Blood plasma was collected at 10 weeks postpartum and analyzed with gas chromatography–mass spectrometry metabolomics.

**Results:** Variations of metabolomic profiles within the PPDS samples were identified. One cluster showed altered kidney function, whereas the other, a metabolic syndrome profile, both previously associated with depression. Five metabolites (glycerol, threonine, 2-hydroxybutanoic acid, erythritol, and phenylalanine) showed higher abundance among women with PPDSs, indicating perturbations in the serine/threonine and glycerol lipid metabolism, suggesting oxidative stress conditions.

**Conclusions:** Alterations in certain metabolites were associated with depressive pathophysiology postpartum, whereas diversity in PPDS physiologies was revealed. Hence, plasma metabolic profiling could be considered in diagnosis and pathophysiological investigation of PPD toward providing clues for treatment. Future studies require standardization of various subgroups with respect to symptom onset, lifestyle, and comorbidities.

## Introduction

Postpartum depression (PPD) affects 1 of 10 women giving birth (1). Poor mother–infant bonding (2), shortened breastfeeding duration (3), and increased suicide risk (4) are just some of many possible consequences of PPD. Furthermore, there is an increased risk of depression for the partner (5) and of behavioral problems for the children (6). However, our understanding about the pathophysiology of the disease, and biological tools for early and accurate diagnosis, remain limited. Advancements on the understanding of the complex underlying pathophysiology could be of great value for accurate and timely diagnosis, prognosis, and treatment, contributing to ameliorating the high societal effects associated with PPD (7).

Evidence of alterations in various biomarkers associated with PPD is increasing (8). The dramatic physical changes involved during pregnancy, including an increased basal metabolic rate (9–11), changes in body fat composition (12, 13), and insulin resistance (14), alterations in various hormonal levels (15–17), inflammatory factors (18–20), levels of neurotransmitters (21), and altered neuro-oxidative and nitrosative stress (22), along with individual sensitivity to such changes, could contribute to increased risk of PPD.

Metabolomics has emerged as a useful tool in psychiatry in recent years, providing a comprehensive view of the metabolic physiology, which combines genetic, epigenetic, and environmental effects (23, 24). As a high-throughput biomolecular analysis, untargeted metabolomics can lead to the identification of discriminatory multi-compound profiles. Monitoring the metabolic physiology of biological systems through the interplay and intervariation of many metabolites in a multivariate way, in combination with the knowledge-based analysis of the metabolite abundances in the context of the metabolic pathways and processes to which they belong, can reveal significance even in subtle quantitative differences without requiring large sample sizes (25). Blood plasma and urine metabolomics have been successfully used in the investigation of other disease pathophysiologies. However, a challenge in psychiatric and neurological diseases is whether plasma and urine metabolomics could be informative of brain physiology [reviewed in (23, 26, 27)], with cerebrospinal fluid profiling expected to be more relevant, but hard to obtain for healthy subjects (24). There are, however, studies that have shown that blood plasma (28), urine (29), or cerebrospinal fluid (30) metabolic profiles could discriminate between subjects affected by psychiatric conditions and healthy participants. A 2016 meta-analysis of 17 articles including 31,880 people associated depression with the metabolic syndrome (31) and a 2020 meta-analysis including 15,000 participants found a discriminatory profile of circulating lipids in depressed patients (32).

For perinatal depression, omic analyses in general and metabolomics in particular are very limited. A study of our group (33) provided a connection between antenatal depression and a plasma metabolic profile rich in fatty acids and/or sugars, but low in branched-chain amino acids (BCAAs), in summer delivery cases. Another study (34) reported higher plasma levels of three triacylglycerol metabolites and lower levels of betaine, citrulline, C5, and C5:1 carnitine to be associated with antenatal depression.

In the case of PPD, some metabolite or metabolite-class targeted analyses have been reported. From these studies, PPD has been associated with alterations in the levels of tryptophan and kynurenine (35, 36), allopregnanolone (37, 38), leptin (39), steroids (40), glutathione–disulfide, adenyl–succinate, and ATP (41).

To our knowledge, only two untargeted metabolomic studies for PPD have been reported to date, both based on urine samples (42, 43). One of them (42) presented a metabolite panel (formate, succinate, 1-methylhistidine, α-glucose, and dimethylamine) for PPD diagnosis, whereas the other (43) found 10 metabolites [alanine, methylmalonic acid, homocysteine, tyrosine, glutaric acid, vanillylmandelic acid, 4-hydroxyhippuric acid, 4-hydroxybenzoic acid, 5-hydroxyindoleacetic acid, 3-(3-hydroxyphenyl)-3, and hydroxypropionic acid] to differentiate between depressed and healthy controls. Still, the current literature, including both targeted and untargeted studies, provide inconclusive results with respect to the metabolic alterations involved in PPD. This could be due to the complexity and heterogeneity of the disease, the sample sizes of the cohorts, the different surrogate tissues analyzed, the time of PPD diagnosis after pregnancy, and/or the difficulty in PPD characterization and severity classification.

The aim of this explorative nested case–control study was to investigate whether blood plasma metabolic profiles could be discriminatory between women with and without PPD symptoms (PPDSs), using untargeted gas chromatography–mass spectrometry (GC-MS) metabolomics, which monitors mainly the primary metabolism.

## Materials and Methods

### Participants

This study is a substudy conducted within the framework of the BASIC project (Biology, Affect, Stress, Imagining, and Cognition), which started in 2009 and finished recruitment in 2018. The BASIC study has been previously described (44). All Swedish-speaking women ≥18 years of age, who were scheduled for a routine ultrasound at Uppsala University Hospital, were invited to participate. After providing informed consent, web-based surveys were answered at approximately gestational weeks 17 and 32, and 6 weeks postpartum (wpp). Included in the surveys were questions on background characteristics and scales including the Edinburgh Postnatal Depression Scale (EPDS) (45). EPDS is a self-reported screening tool commonly used among the Swedish population and has a validated cutoff of 12 points, with higher values associated with higher severity of the disease, and sensitivity of 96% and specificity of 49% (46).

A subgroup of women participating in the BASIC project, who scored either ≥12 or 1–8 at the EPDS filled in at 6 wpp, was invited to the research laboratory 10 wpp for further examinations. The examinations included repetition of the EPDS, the Mini-International Neuropsychiatric Interview (MINI) version 6.0, sections A. Depression and N. Generalized anxiety disorder, and blood sampling. Cases were defined as EPDS scores 12–30 (*n* = 12) at 6 wpp and controls as EPDS 1–8 (*n* = 12) at all the time points during pregnancy and postpartum included in the BASIC study. Some of the cases (*n* = 6) did not still score ≥12 points at the EPDS 10 wpp. Furthermore, controls were eligible if reporting no history of depressive episodes according to the MINI interview. As well-defined lifestyle and family environment are important in psychiatric and PPD research, the 24 women included were selected based on strict inclusion criteria: (a) 26–39 years of age, (b) body mass index (BMI) between 20.0 and 29.9 kg/m^2^, (c) non-smokers prior and during pregnancy and at time of blood sampling, (d) parity ≤ 4, (e) glucose levels <6.8 mmol/L during pregnancy, (f) no medication except antidepressants (for cases) or levothyroxine at time of invitation and sample collection, (g) breastfeeding, (h) no pregnancy complications including diabetes, (i) no twin pregnancies, (j) blood loss during delivery <1,000 mL, and (k) no unhealed lacerations at 10 wpp. These criteria were applied with the purpose of examining a very well-defined cohort with respect to PPD, based on the medical records of the subjects, any confounding factors, and the knowledge that we had gained from our previous study investigating antenatal depressive symptoms (33). All participating women had been fasting overnight, and visits were scheduled in the morning, mean time 8:54 am. Previously, seasonal variations in metabolites of pregnant women had been reported (33). Therefore, only women who donated blood during the period April to September, that is, during the summer season, were included in this substudy.

### Ethical Considerations

Written informed consent was obtained from all participants when entering the BASIC study, as well as prior to any sampling or testing at the research laboratory. The study protocol was approved by the Regional Ethical Review Board of Uppsala, Sweden (Dnr 2009/171).

### Sample Collection

Venous blood samples were collected and prepared as described by Brann et al. (20). Samples were later thawed and two 100 μL plasma aliquots per participant were shipped on dry ice to FORTH/ICE-HT for the metabolomic analysis.

### Metabolomic Data Acquisition and Normalization

Metabolite extraction, metabolic profile acquisition, metabolite identification, and normalization protocols were applied as in Henriksson et al. (33), with the addition of 5 μg ribitol (AlfaAesar, Germany) to each 100 μL plasma aliquot as internal standard, instead of 0.05 μg ribitol in Henriksson et al. (33). Peak identification was based on the commercial NIST and our in-house MESBL peak library (47, 48). Eighty-five metabolite derivative peaks were identified in at least one of the acquired profiles. Metabolite derivative abundances were estimated from the relative peak area (RPA) of the marker ion of each metabolite derivative with respect to the peak area of the internal standard ribitol ion 319. Data validation, normalization, and filtering of the low confidence measurements and inconsistently detected metabolite derivatives [>25% mean CoV between technical replicates; >30% mean CoV between aliquots (48–50)] were carried out using our group's GC-MS metabolomic analysis software M-IOLITE (http://miolite2.iceht.forth.gr) (47). After metabolite derivative combination, normalization, and filtering, the normalized profiles involved 38 metabolites. Metabolic profile of each aliquot was estimated as mean of normalized and filtered profiles of all its technical replicates. Metabolic profile of each sample was estimated as the mean metabolic profile of its aliquots. Final normalized dataset used for further analysis is provided in Supplementary Table 1.

### Statistical Analysis

#### Demographic, Medical, and Questionnaire Data

Background characteristics were analyzed using the Statistical Package for the Social Sciences version 26. For univariate analyses, Student *t*-test and Mann–Whitney *U*-test were applied for continuous variables as suited, whereas the χ^2^ test was applied for categorical variables. Statistical significance threshold was set at *p* < 0.05.

#### Primary Multivariate Statistical Analysis of the Metabolomic Dataset

For primary analysis, hierarchical clustering (HCL), principal component analysis (PCA), and significance analysis for microarrays (SAM) algorithms were implemented in version 4.9 of the omics data analysis TM4/MeV software (51). Analysis was performed with missing values not imputed. Metabolites identified by SAM with concentration significantly higher or lower in a set of metabolic profiles compared to another are referred to as positively or negatively significant metabolites of the particular comparison, respectively. Analysis was based on the standardized metabolomic dataset.

The standardized RPA of a metabolite M in the profile j, $stRPA_{M}^{j}$, equals to:

$$\begin{array}{l} \text{stRP}{\text{A}}_{\text{M}}^{\text{j}}=\frac{\text{RP}{\text{A}}_{\text{M}}^{\text{j}}-\bar{\text{RP}{\text{A}}_{\text{M}}}}{\text{S}{\text{D}}_{\text{RP}{\text{A}}_{\text{M}}}} \end{array}$$

where $RPA_{M}^{j}$, $\bar{RPA_{M}}$, and *SD*_*RP*_*A*__*M*__ depict, respectively, the RPA of metabolite *M* in profile *j*, the mean RPA of metabolite *M*, and its standard deviation in all profiles.

In SAM, the threshold of significance (δ) is selected as the largest for the false discovery rate (FDR) median to be < 10% or that corresponding to the smallest FDR median, if the latter is >10%, as specified in each case.

#### Sensitivity Analyses

Based on previous experience, to identify discriminatory metabolites in possible subgroups within the groups, sensitivity analyses (excluding very metabolically diverse individuals in comparison to the rest of their group) were carried out using SAM algorithms. Selection criteria for the significance threshold were the same as described for the primary analysis.

## Results

### Demographic, Medical, and Questionnaire Data

Univariate analyses of background characteristics showed only a slight difference in the number of days from parturition until the day of blood sampling between the controls and women with PPDSs (apart from the expected difference in EPDS scores, previous depressive episodes, and use of antidepressants, which were the guiding variables in the control/case definition) (Table 1).

**Table 1:** Background characteristics of women with and without postpartum depressive symptoms.

**Variable**	**Control (*n* = 12)**	**PPDS (*n* = 12)**	***p*-value**
EPDS 6 wpp, median (IQR)	4.0 (0.8)	16.0 (4.8)	0.000
EPDS 10 wpp, median (IQR)	3.0 (2.8)	11.5 (7.5)	0.000
MINI-defined ongoing depression, *n* (%)	0 (0)	3 (25)	—
MINI-defined previous depression, *n* (%)	0 (0.0)	11 (91.6)	—
MINI-defined GAD, *n* (%)	0 (0.0)	1 (8.3)	—
SSRI-use, *n* (%)	0 (0)	4 (33.3)	0.028
Age, mean (SD), years	32.6 (3.5)	32.3 (3.7)	0.799
BMI, median (IQR), kg/m^2^	24.1 (1.1)	25.0 (6.1)	0.449
University education, *n* (%)	9 (81.8)	9 (81.8)	1.000
Breastfeeding 6 vpp, *n* (%)			0.059
Breastfeeding only	11 (91.7)	7 (58.3)	
Breastfeeding and formula	1 (8.3)	5 (41.7)	
Levothyroxine treatment, *n* (%)	2 (16.7)	4 (33.3)	0.346
Nulliparous, *n* (%)	4 (33.3)	5 (41.7)	0.673
Days from parturition, median (IQR)	74 (4.8)	79 (8.0)	0.020

*PPDSs, postpartum depressive symptoms; EPDS, Edinburgh Postnatal Depression Scale; IQR, interquartile range; SSRI, selective serotonin reuptake inhibitor; SD, standard deviation; BMI, body mass index; MINI, Mini-International Neuropsychiatric Interview; GAD, generalized anxiety disorder*.

Of the 12 women who reported depressive symptoms according to the EPDS at 6 wpp, six (participants 9, 13, 17, 26, 34, and 35) did not pass the 12-point threshold in the EPDS filled at 10 wpp. However, these women were still included in the PPDS group. Nevertheless, one woman with EPDS < 12 at 10 wpp (participant 26) did meet the criteria for ongoing depression at the MINI interview.

### Primary Metabolomic Data Analysis

**Figure 1:**
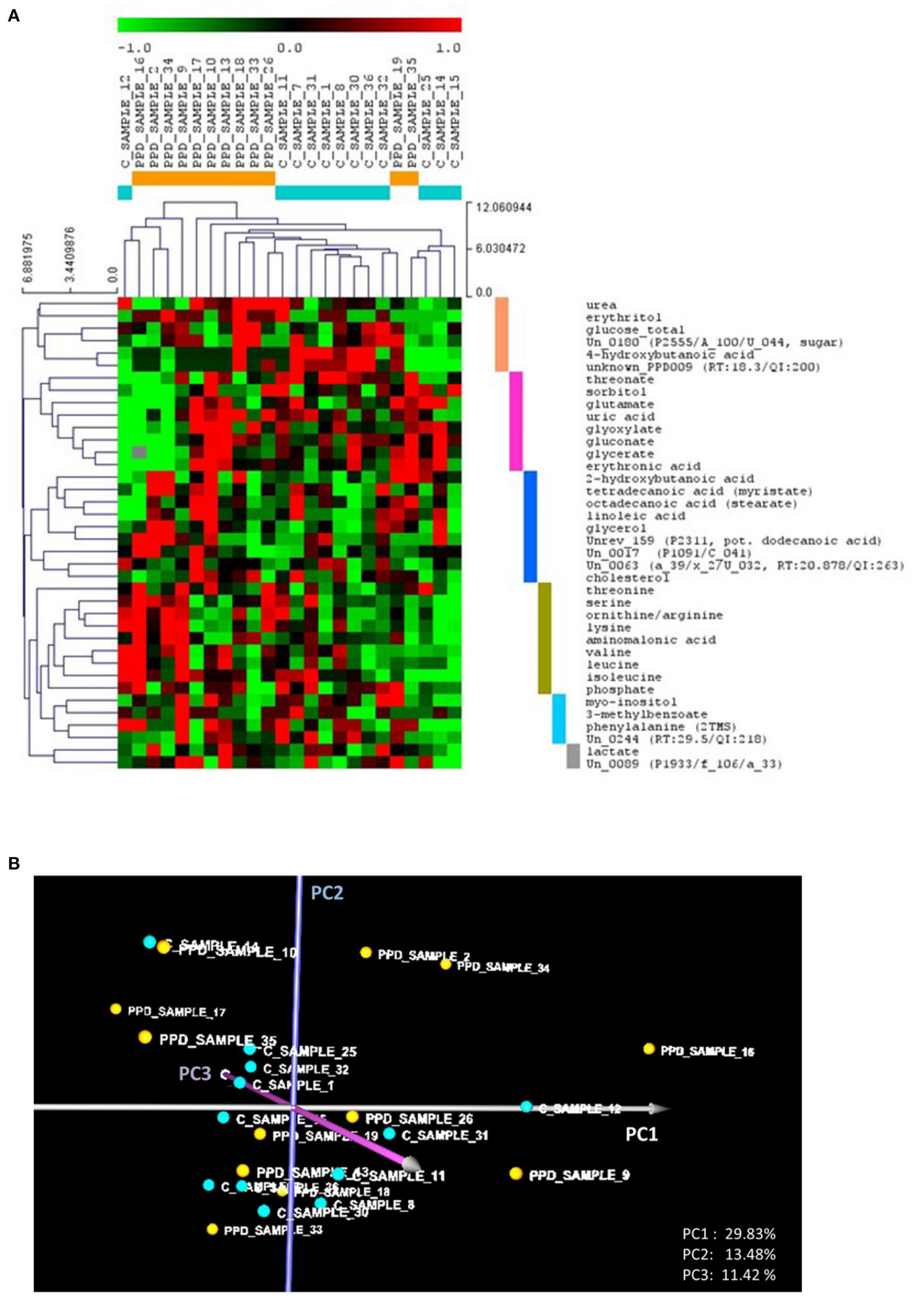
**(A)** Hierarchical trees for both samples and metabolites based on hierarchical clustering (HCL) analysis of the standardized metabolic profiles (Euclidean distance). The colored bars on the right side of the HCL heat map depict the identified metabolite clusters, discussed in the text. **(B)** The PCA graph of the standardized metabolic profiles, depicting the relative position of the controls compared to the PPDS sample profiles. In both **(A,B)**, the control and PPDS sample profiles are colored, respectively, light blue and orange. PC_i_ refers to principal component axis *i*.

The standardized metabolomic dataset (Supplementary Table 1) was analyzed using HCL and PCA (Figures 1A,B). Observed metabolite clusters in the HCL heat map are color coded. HCL indicated differences in the metabolic profiles of the controls and PPDS cases that could discriminate between most of them. However, there were distinct subclusters within the PPDS samples. Both HCL and PCA indicated a distinct cluster that comprised PPDS samples 2, 9, 16, and 34, and control sample 12 [the left branch of the heat map in Figure 1A and the right part of the PCA graph (positive PC1 values) in Figure 1B]. According to the heat map, the particular cluster was characterized by high abundances in the “olive green” metabolite cluster, including mainly the amino acids threonine, serine, ornithine/arginine, valine, leucine, isoleucine, lysine, and the aminomalonic acid, combined, though, with distinctly low abundances in the “purple” metabolite cluster, including threonate, sorbitol, glutamate, uric acid, glyoxylate, gluconate, glycerate, and erythronate. The differences in the profile of participant 9 compared to other samples in this cluster were higher abundances of the metabolites in the “purple” cluster, and high abundances of the metabolites myo-inositol, 3-methylbenzoate, phenylalanine, and the unannotated metabolite Un_0244 of the “light blue” cluster.

Based on the heat map, the rest of the PPDS samples, including samples 17, 10, 13, 18, 33, 26, 19, and 35, showed, in controversy to the first cluster, no significant differences in the abundance of the metabolites of the “olive green” cluster, but higher abundances in the “purple” cluster metabolites, compared to the controls (excluding participant 12). In addition, this PPDS sample group shows higher abundances in the “blue” (“fatty acid”) metabolite cluster, including cholesterol, linoleic acid, stearate, myristate, putative lauric acid, 2-hydroxybutanoic acid, and glycerol, and/or the “pink” cluster, including urea, erythritol, 4-hydroxybutanoic acid, and glucose, compared to the controls (excluding participant 12).

Taking into consideration the loads of the PC axes, the observations from the HCL analysis are supported by the position of the profiles in the PCA graph. Positive values on *PC1* correspond to high abundances of the “olive green” cluster metabolites combined with low abundances of the “purple” cluster metabolites. Positive values on *PC2* correspond to high abundances of the “blue” (fatty acid) cluster metabolites combined with low abundances of “pink” cluster metabolites. Lastly, positive values on *PC3* correspond mainly to low glucose and cholesterol combined with high abundances of threonate, ornithine/arginine, and the “light blue” cluster metabolites myo-inositol, 3-methylbenzoate and phenylalanine. Accordingly, negative values in the respective axes correspond to the opposite profile discussed for each axis above. The further a profile is from the PCA graph origin, the more it tends to be associated with a PPDS participant's sample.

Multivariate significance analysis based on the SAM method of the complete set of the control and PPDS profiles indicated that the total abundance of the 38 metabolites included in the analysis is on average larger in the PPDSs than in the controls. Additionally, 18 metabolites were positively significant with no metabolite identified as negatively significant in the PPDS profiles compared to the control profiles, although there was a high number of false positives (i.e., 5.5 metabolites or 30.7% FDR-median at the strictest threshold of significance that provided any results). The complete list of the 18 positively significant metabolites in decreasing order of significance is shown in Table 2.

**Table 2:** List of positively and negatively significant metabolites in decreasing order of significance in the primary and the two sensitivity analyses based on the SAM method.

**Primary analysis (PPDS) vs. (controls)**	**Sensitivity analysis 2A (PPDS samples 2,9,16, 34) vs. (controls but sample 12)**		**Sensitivity analysis 2B (PPDS samples 17, 10, 13, 18, 33, 26, 19, 35) vs. (controls but sample 12)**
**Positively significant**	**Positively significant**	**Negatively significant**	**Positively significant**
1. glycerol	1. aminomalonic acid	1. gluconate	1. uric acid
2. threonine	2. lysine	2. glycerate	2. stearate
3.2-hydroxybutanoic acid	3. dodecanoic (lauric) acid putative	3. glyoxylate	3. urea
4. erythritol	4. ornithine+arginine	4. erythronate	4.2-hydroxybutanoic acid
5. aminomalonic acid	5. serine	5. sorbitol	5. linoleic acid
6. Un_0089 (P1933/f_106/a_33)	6. valine		6. threonine
7. phenylalanine	7. leucine		7. erythronate
8. stearate	8. glycerol		8. cholesterol
9. linoleic acid	9. isoleucine		9. glycerol
10. serine	10. Un_0017 (P1091/C_041)		10. Un_0089 (P1933/f_106/a_33)
11. ornithine+arginine	*FDR-median = 0%*		11. phenylalanine
12. lysine	11. erythritol		12. erythritol
13. lactate	12. phenylalanine		13. glyoxylate
14. Un_0017 (P1091/C_041)	*FDR-median = 2.48%*		14. lactate
15. uric acid	13. threonine	6. threonate	*FDR-median = 16.92%*
16. myristate	*FDR-median = 4.40%*		
17. myo-inositol	14. myo-inositol		
18. cholesterol	15.3-methylbenzoate		
*FDR-median = 30.7%*	*FDR-median = 6.00%*		
19. isoleucine	16. Un_0089	7. urea	
*FDR-median = 33.2%*	17.2-hydroxybutanoic acid	8. uric acid	
		9. glutamate	
	*FDR-median *=* 9.72%*		

*FDR, false discovery rate; Un_, Unannotated metabolite (in parenthesis previously used IDs in published studies); positively significant metabolite in Group B vs. Group A means that the metabolite is of higher abundance in Group B compared to Group A; negatively significant metabolite in Group B vs. Group A means that the metabolite is of lower abundance in Group B compared to Group A*.

### Sensitivity Metabolomic Data Analysis

Two sensitivity SAM analyses were performed, comparing separately the different PPDS clusters observed in the primary analysis. Excluding control sample 12, which, based on HCL, could have a physical condition not yet aware of, comparisons of (a) the PPDS subcluster of samples 2, 9, 16, and 34 with all controls (analysis 2A) and (b) the rest of the PPDS samples with all the controls (analysis 2B) were performed.

In the SAM analysis 2A, at the largest significance threshold for which FDR median is < 10%, 26 of the 38 metabolites were identified as statistically different between the two groups (FDR-median = 9.72% or 2.5 metabolites). It is noted that in this analysis, 15 metabolites were identified as distinctly differential at FDR-median = 0%. Among the 26 metabolites, 17 were positively and 9 negatively significant in the PPDS subcluster compared to the controls (10 and 5 were identified in the respective groups at FDR-median = 0%). The significant metabolites, shown in Table 2 in decreasing order of significance, confirm the observations from the HCL and PCA analyses (Figures 1A,B). The small FDR supports the distinctness of the metabolic profile of the PPDS subgroup (participants 2, 9, 16, and 34) compared to the controls.

In the SAM analysis 2B, where the rest of the PPDS samples were compared with all controls but sample 12, 14 positively and none negatively significant metabolites were identified (with FDR-median = 16.92% or 2.4 out of the 14 metabolites). The significant metabolites of 2B analysis are shown in Table 2.

Five metabolites, glycerol, threonine, 2-hydroxybutanoic acid, erythritol, and phenylalanine, were identified as positively significant in both sensitivity (2A and 2B) analyses and the overall primary comparison between PPDS and control sample profiles.

## Discussion

Metabolomic analyses indicated discriminatory differences between the metabolic profiles of most controls and PPDS samples. However, there were distinct subclusters within the PPDS samples, which need to be taken into consideration if high-risk metabolic biomarkers for PPD are to be accurately identified. It appears that multiple different metabolic profiles are associated with PPDS subtypes, supporting the need to standardize PPD molecular phenotyping with respect to time of onset, time of profiling, and/or symptom subgroups, to enable accurate and sensitive diagnosis and biomarker detection. Although further validation in a larger cohort is necessary, our findings are supported by previous studies suggesting heterogeneity within PPD (52–54).

The two sensitivity analyses suggested two distinct PPDS clusters, one with high abundance of amino acids, fatty acids, and glycerol–phospholipid metabolism intermediates, and low abundance of sugars and sugar acids, compared to the controls. Lower abundance of sugars combined with higher of BCAAs is commonly considered as a “favorable” metabolic profile. The combination of higher ornithine+arginine to urea ratio in this cluster of PPDS samples suggests an aberrant urea cycle compared to the controls (55). During pregnancy, the glomerular filtration rate increases, leading to decreased urea and uric acid values (56). We speculate that these women might not have been able to regulate the filtration rate back to a non-pregnant state, perhaps due to the impact of oxidative stress on kidney function even postpartum (57). Even though GC-MS cannot differentiate between arginine and ornithine, these findings combined with the urea measurement may indicate lower arginase activity (i.e., higher abundance of arginine and lower of ornithine) in the PPDS samples compared to the controls, as both urea and ornithine are products of the arginase-catalyzed reaction of the urea cycle. Such profile has been associated with insulin resistance and type 2 diabetes mellitus (58); arginase is regulated by insulin. Decreased urea has been associated with increased blood pressure (BP) (59), and dysregulation of BP has in turn been linked to depression [reviewed in (60)]. Unfortunately, BP measurements were not included in the assessment of the participants in this study, but may be considered regularly in studies examining depression. It is noted that in this study none of the participants had a history of hypertension before or during pregnancy. Furthermore, aminomalonic acid was identified as the metabolite with the highest positive difference in the PPDS samples compared to the controls. Production of aminomalonic acid is considered associated with oxidative damage (61, 62) and has previously been used in a potential biomarker panel for depression and anxiety outside the perinatal period (63).

The second group of PPDS samples included in the second sensitivity analysis shows what is considered a typical “unfavorable” metabolic profile, with higher levels of metabolites comprising mostly fatty and sugar acids. This type of profile is seen among individuals with metabolic syndrome (64), which has been associated with depression (31). Notably, the abundance of the metabolites uric acid, urea, erythronate, and glyoxylate are identified as lower in one PPDS cluster and higher in the other compared to the controls, again indicating different PPD subtypes in need of further investigation.

The control participant, who presented a pathological metabolic profile, was excluded from the sensitivity analyses as a consequence of having a very diverse profile compared to the rest of the controls. However, we could not confirm any pathology recorded in her medical records. Along with the two metabolically different subgroups of PPDS samples, two “metabolically healthy” depression-positive cases (samples 19 and 35, see Figure 1A) were identified from our multivariate analyses. One was the PPDS participant without any previous depressive episodes, thus she may not have yet developed a relevant metabolic fingerprint. The other participant was the PPDS participant with the lowest EPDS score in both 6 wpp (EPDS = 12) and 10 wpp (EPDS = 6), hence she had the mildest depressive symptoms and metabolic profiling classified her with the controls at 10 wpp. These results support the hypothesis that not all PPDS cases have the same pathophysiology (53), and only some have a distinct metabolic signature.

Moreover, five of the women with PPDSs at 6 wpp did not present depressive symptoms at 10 wpp; two of these samples were found in the first cluster, whereas the other three in the second. It is unclear whether loss of symptoms depends on the location where the EPDS is answered [at the research laboratory (10 wpp) or at other location (6 wpp)], or whether the symptoms were mild and mostly resolved within 10 wpp. These findings highlight the importance of standardization of the time of diagnosis and sample collection.

Five metabolites, glycerol, threonine, 2-hydroxybutanoic acid, erythritol, and phenylalanine, were identified as positively significant in both PPDS clusters and the overall comparison between PPDS and control profiles. These five were not included in neither the panel of metabolites previously identified by Lin et al. (42) nor the findings of Zhang et al. (43), but all five have some association with oxidative stress conditions: one of the areas of interest in research of PPD (22). Glycerol, an alcohol released into the bloodstream when stored fat is used in the energy metabolism, has been shown in animal studies to induce oxidative stress (65). Threonine, an essential amino acid important for the nervous system, is transferred across the blood brain barrier and is converted to glycine. Glycine metabolism is one of the pathways found to be altered in rat models of depression (66), and animal studies have reported that accumulation of threonine affects the neurotransmitter balance (67). The early indicator of insulin resistance, 2-hydroxybutanoic acid (68), can be catabolized from threonine (69). Alterations in threonine and the threonine catabolism intermediate 2-hydroxybutanoic acid indicate perturbations in the serine/threonine metabolism associated with oxidative stress. Increased levels of 2-hydroxybutanoic acid have been observed in women with antenatal depression (33). Erythritol is a sugar alcohol used as a food additive and naturally found in fruits. Central adiposity, included as a factor in the definition of the metabolic syndrome (70), is associated with increased levels of erythritol (71). Furthermore, animal studies have shown erythritol to induce oxidative stress (72), and increased erythritol levels in the depressed patients could be a response to increased oxidative stress. Phenylalanine, a precursor of the neurotransmitters dopamine and norepinephrine, has been shown to increase oxidative stress in rats (73). When accumulated in the body, phenylalanine is causatively associated with neuropsychological dysfunction and depressed mood (74).

The strict inclusion criteria implemented in this study provide a homogenous, generally healthy (apart from depression) study group with no extremes concerning age or BMI. Analyses of background characteristics showed no differences between the controls and the PPDS group aside from days from parturition until blood collection, EPDS scores, and use of antidepressants, suggesting otherwise comparable study groups. Time of blood sample collection (10 wpp) is considered as a stable time period in terms of hormonal levels (75). As time-of-day variation of metabolic rates has been observed (76, 77), a strength of this study is that all samples were collected during the morning hours. Furthermore, as seasonal variations in metabolic profiles have been observed in women with antenatal depressive symptoms (33), the inclusion of only summer births should be seen as a strength of the study, eliminating any seasonal effect biases. This, of course, has an impact on generalizability of the results, and future studies including the winter months are merited. Furthermore, standardized collection of material for metabolomic studies is crucial, and the samples collected in this study came from women who were fasting overnight as recommended (78).

Our results further support multivariate statistical and data mining methods as a state-of-the-art approach to analyze the omic profiles of complex pathophysiologies, such as PPD (79, 80), and the importance of interpreting the differential biomolecules in the context of interconnected pathways and not as isolated individual biomarkers. Furthermore, our results provide some suggestions about the underlying pathophysiology. However, the complexity and heterogeneous pathophysiology of perinatal depression complicate the discovery of metabolic biomarkers. Future studies need to be implemented in larger cohorts, including patients with severe clinical depression, with consistently defined subgroups with respect to symptom onset, lifestyle, and comorbidities (81). Moreover, integration of different omics is desirable for comprehensive molecular phenotyping.

## Limitations

There are some limitations to the current study. Because of strict inclusion criteria, the sample size was relatively small. Furthermore, sampling has been limited to older than average and more educated women. The generalizability of the conclusions requires broader sampling.

## Conclusion

In this exploratory metabolomics study, we were not able to define a unique metabolic profile of women with PPDSs. However, we identified two clusters of women within the PPDSs presenting different profiles. One cluster appeared to have altered kidney function, whereas the other showed a metabolic syndrome–related profile, both previously associated with depression. These findings need to be further validated in future larger studies. As depression is a heterogeneous diagnosis with different symptoms associated with different metabolic pathways, our results support the need to continue examining potential distinct patient groups within the PPD spectrum.

## Data Availability Statement

The raw data supporting the conclusions of this article will be made available by the authors, without undue reservation.

## Ethics Statement

The studies involving human participants were reviewed and approved by Regional Ethical Review Board of Uppsala, Sweden (Dnr 2009/171). The patients/participants provided their written informed consent to participate in this study.

## Author Contributions

AS and MK conceptualized and designed the study. EB, CM, M-KI, AS, and MK performed the analyses and interpreted the results together with EF, FP, and GC. EB and HH were responsible for data acquisition from participants. EB, EF, AS, and MK wrote the draft of the manuscript. HH, CM, M-KI, FP, and GC revised the manuscript critically for important intellectual content. All authors contributed to the article and approved the submitted version.

## Conflict of Interest

The authors declare that the research was conducted in the absence of any commercial or financial relationships that could be construed as a potential conflict of interest.

## References

[1] Woody CA Ferrari AJ Siskind DJ Whiteford HA Harris MG. A systematic review and meta-regression of the prevalence and incidence of perinatal depression. J Affect Disord. (2017) 219:86–92. 10.1016/j.jad.2017.05.003 28531848

[2] Dubber S Reck C Muller M Gawlik S. Postpartum bonding: the role of perinatal depression, anxiety and maternal-fetal bonding during pregnancy. Arch Womens Ment Health. (2015) 18:187–95. 10.1007/s00737-014-0445-4 25088531

[3] Figueiredo B Canario C Field T. Breastfeeding is negatively affected by prenatal depression and reduces postpartum depression. Psychol Med. (2014) 44:927–36. 10.1017/S0033291713001530 23822932

[4] Esscher A Essen B Innala E Papadopoulos FC Skalkidou A Sundstrom-Poromaa I. Suicides during pregnancy and 1 year postpartum in Sweden, 1980-2007. Br J Psychiatry. (2016) 208:462–9. 10.1192/bjp.bp.114.161711 26494874

[5] Paulson JF Bazemore SD. Prenatal and postpartum depression in fathers and its association with maternal depression: a meta-analysis. JAMA. (2010) 303:1961–9. 10.1001/jama.2010.605 20483973

[6] Agnafors S Sydsjo G Dekeyser L Svedin CG. Symptoms of depression postpartum and 12 years later-associations to child mental health at 12 years of age. Matern Child Health J. (2013) 17:405–14. 10.1007/s10995-012-0985-z 22466717

[7] Bauer A Knapp M Parsonage M. Lifetime costs of perinatal anxiety and depression. J Affect Disord. (2016) 192:83–90. 10.1016/j.jad.2015.12.005 26707352

[8] O'hara MW Wisner KL. Perinatal mental illness: definition, description and aetiology. Best Pract Res Clin Obstet Gynaecol. (2014) 28:3–12. 10.1016/j.bpobgyn.2013.09.002 24140480 PMC7077785

[9] Forsum E Sadurskis A Wager J. Resting metabolic rate and body composition of healthy Swedish women during pregnancy. Am J Clin Nutr. (1988) 47:942–7. 10.1093/ajcn/47.6.942 3376909

[10] Chihara H Otsubo Y Araki T. Resting energy expenditure in pregnant Japanese women. J Nippon Med School. (2002) 69:373–5. 10.1272/jnms.69.373 12187370

[11] Shinagawa S Suzuki S Chihara H Otsubo Y Takeshita T Araki T. Maternal basal metabolic rate in twin pregnancy. Gynecol Obstet Invest. (2005) 60:145–8. 10.1159/000086132 15925892

[12] Lederman SA Paxton A Heymsfield SB Wang J Thornton J Pierson N JR. Body fat and water changes during pregnancy in women with different body weight and weight gain. Obstet Gynecol. (1997) 90:483–8. 10.1016/S0029-7844(97)00355-4 9380301

[13] Azizian H Kramer JK Phillips SM. First direct body fat content measurement during pregnancy using Fourier transform near-infrared spectroscopy. Appl Spectrosc. (2014) 68:379–82. 10.1366/13-07256 24666956

[14] Sonagra AD Biradar SM K D Murthy DSJ. Normal pregnancy- a state of insulin resistance. J Clin Diagn Res. (2014) 8:CC01-03. 10.7860/JCDR/2014/10068.5081 25584208 PMC4290225

[15] Jung C Ho JT Torpy DJ Rogers A Doogue M Lewis JG. A longitudinal study of plasma and urinary cortisol in pregnancy and postpartum. J Clin Endocrinol Metab. (2011) 96:1533–40. 10.1210/jc.2010-2395 21367926

[16] Iliadis S Sylvén S Jocelien O Hellgren C Hannefors A.-K Elfström D. Corticotropin-releasing hormone and postpartum depression: a longitudinal study. Psychoneuroendocrinology. (2015) 61:61. 10.1016/j.psyneuen.2015.07.556

[17] Schiller CE Meltzer-Brody S Rubinow DR. The role of reproductive hormones in postpartum depression. CNS Spectr. (2015) 20:48–59. 10.1017/S1092852914000480 25263255 PMC4363269

[18] Maes M Lin AH Ombelet W Stevens K Kenis G De Jongh R. Immune activation in the early puerperium is related to postpartum anxiety and depressive symptoms. Psychoneuroendocrinology. (2000) 25:121–37. 10.1016/S0306-4530(99)00043-8 10674277

[19] Corwin EJ Pajer K Paul S Lowe N Weber M Mccarthy DO. Bidirectional psychoneuroimmune interactions in the early postpartum period influence risk of postpartum depression. Brain Behav Immun. (2015) 49:86–93. 10.1016/j.bbi.2015.04.012 25937051 PMC4567438

[20] Brann E Fransson E White RA Papadopoulos FC Edvinsson A Kamali-Moghaddam M. Inflammatory markers in women with postpartum depressive symptoms. J Neurosci Res. (2018) 98:1309–21. 10.1002/jnr.24312 30252150

[21] Sanjuan J Martin-Santos R Garcia-Esteve L Carot JM Guillamat R Gutierrez-Zotes A. Mood changes after delivery: role of the serotonin transporter gene. Br J Psychiatry. (2008) 193:383–8. 10.1192/bjp.bp.107.045427 18978318

[22] Roomruangwong C Anderson G Berk M Stoyanov D Carvalho AF Maes M. A neuro-immune, neuro-oxidative and neuro-nitrosative model of prenatal and postpartum depression. Prog Neuropsychopharmacol Biol Psychiatry. (2018) 81:262–74. 10.1016/j.pnpbp.2017.09.015 28941769

[23] Guest PC Guest FL Martins-De Souza D. Making sense of blood-based proteomics and metabolomics in psychiatric research. Int J Neuropsychopharmacol. (2016) 19:1–10. 10.1093/ijnp/pyv138 26721951 PMC4926797

[24] Vasilopoulou CG Margarity M Klapa MI. Metabolomic analysis in brain research: opportunities and challenges. Front Physiol. (2016) 7:183. 10.3389/fphys.2016.00183 27252656 PMC4878281

[25] Billoir E Navratil V Blaise BJ. Sample size calculation in metabolic phenotyping studies. Brief Bioinform. (2015) 16:813–9. 10.1093/bib/bbu052 25600654

[26] Huang TL Lin CC. Advances in biomarkers of major depressive disorder. Adv Clin Chem. (2015) 68:177–204. 10.1016/bs.acc.2014.11.003 25858873

[27] Nedic Erjavec G Konjevod M Nikolac Perkovic M Svob Strac D Tudor L Barbas C. Short overview on metabolomic approach and redox changes in psychiatric disorders. Redox Biol. (2018) 14:178–86. 10.1016/j.redox.2017.09.002 28942195 PMC5609866

[28] Zheng H Zheng P Zhao L Jia J Tang S Xu P. Predictive diagnosis of major depression using NMR-based metabolomics and least-squares support vector machine. Clin Chim Acta. (2017) 464:223–7. 10.1016/j.cca.2016.11.039 27931880

[29] Zheng P Wang Y Chen L Yang D Meng H Zhou D. Identification and validation of urinary metabolite biomarkers for major depressive disorder. Mol Cell Proteomics. (2013) 12:207–14. 10.1074/mcp.M112.021816 23111923 PMC3536901

[30] Pan LA Martin P Zimmer T Segreti AM Kassiff S Mckain BW. Neurometabolic disorders: potentially treatable abnormalities in patients with treatment-refractory depression and suicidal behavior. Am J Psychiatry. (2017) 174:42–50. 10.1176/appi.ajp.2016.15111500 27523499 PMC10171090

[31] Ghanei Gheshlagh R Parizad N Sayehmiri K. The relationship between depression and metabolic syndrome: systematic review and meta-analysis study. Iran Red Crescent Med J. (2016) 18:e26523. 10.5812/ircmj.26523 27621928 PMC5003061

[32] Bot M Milaneschi Y Al-Shehri T Amin N Garmaeva S Onderwater GLJ. Metabolomics profile in depression: a pooled analysis of 230 metabolic markers in 5283 cases with depression and 10,145 controls. Biol Psychiatry. (2020) 87:409–18. 10.1016/j.biopsych.2019.08.016 31635762 PMC11921392

[33] Henriksson HE Malavaki C Brann E Drainas V Lager S Iliadis SI. Blood plasma metabolic profiling of pregnant women with antenatal depressive symptoms. Transl Psychiatry. (2019) 9:204. 10.1038/s41398-019-0546-y 31444321 PMC6707960

[34] Mitro SD Larrabure-Torrealva GT Sanchez SE Molsberry SA Williams MA Clish C. Metabolomic markers of antepartum depression and suicidal ideation. J Affect Disord. (2020) 262:422–8. 10.1016/j.jad.2019.11.061 31744743 PMC6917910

[35] Veen C Myint AM Burgerhout KM Schwarz MJ Schutze G Kushner SA. Tryptophan pathway alterations in the postpartum period and in acute postpartum psychosis and depression. J Affect Disord. (2016) 189:298–305. 10.1016/j.jad.2015.09.064 26454336

[36] Wang S Quan C Tan Y Wen S Zhang J Duan K. Correlation between kynurenine metabolites and postpartum depression. Zhong Nan Da Xue Xue Bao Yi Xue Ban. (2018) 43:725–31. 10.11817/j.issn.1672-7347.2018.07.005 30124207

[37] Hellgren C Comasco E Skalkidou A Sundstrom-Poromaa I. Allopregnanolone levels and depressive symptoms during pregnancy in relation to single nucleotide polymorphisms in the allopregnanolone synthesis pathway. Horm Behav. (2017) 94:106–13. 10.1016/j.yhbeh.2017.06.008 28666923

[38] Osborne LM Gispen F Sanyal A Yenokyan G Meilman S Payne JL. Lower allopregnanolone during pregnancy predicts postpartum depression: an exploratory study. Psychoneuroendocrinology. (2017) 79:116–21. 10.1016/j.psyneuen.2017.02.012 28278440 PMC5420429

[39] Skalkidou A Sylven SM Papadopoulos FC Olovsson M Larsson A Sundstrom-Poromaa I. Risk of postpartum depression in association with serum leptin and interleukin-6 levels at delivery: a nested case-control study within the UPPSAT cohort. Psychoneuroendocrinology. (2009) 34:1329–37. 10.1016/j.psyneuen.2009.04.003 19427131

[40] Parizek A Mikesova M Jirak R Hill M Koucky M Paskova A. Steroid hormones in the development of postpartum depression. Physiol Res. (2014) 63:S277–82. 10.33549/physiolres.932788 24908233

[41] Papadopoulou Z Vlaikou AM Theodoridou D Komini C Chalkiadaki G Vafeiadi M. Unraveling the serum metabolomic profile of post-partum depression. Front Neurosci. (2019) 13:833. 10.3389/fnins.2019.00833 31507354 PMC6716353

[42] Lin L Chen XM Liu RH. Novel urinary metabolite signature for diagnosing postpartum depression. Neuropsychiatr Dis Treat. (2017) 13:1263–70. 10.2147/NDT.S135190 28546751 PMC5436788

[43] Zhang L Zou W Huang Y Wen X Huang J Wang Y. A preliminary study of uric metabolomic alteration for postpartum depression based on liquid chromatography coupled to quadrupole time-of-flight mass spectrometry. Dis Markers. (2019) 2019:4264803. 10.1155/2019/4264803 31178941 PMC6507152

[44] Axfors C Brann E Henriksson HE Hellgren C Kunovac Kallak T Fransson E. Cohort profile: the Biology, Affect, Stress, Imaging and Cognition (BASIC) study on perinatal depression in a population-based Swedish cohort. BMJ Open. (2019) 9:e031514. 10.1136/bmjopen-2019-031514 31641004 PMC6830667

[45] Cox JL Holden JM Sagovsky R. Detection of postnatal depression. Development of the 10-item Edinburgh Postnatal Depression Scale. Br J Psychiatry. (1987) 150:782–6. 10.1192/bjp.150.6.782 3651732

[46] Wickberg B Hwang CP. The Edinburgh Postnatal Depression Scale: validation on a Swedish community sample. Acta Psychiatr Scand. (1996) 94:181–4. 8891084 10.1111/j.1600-0447.1996.tb09845.x

[47] Maga-Nteve C Klapa MI. Streamlining GC-MS metabolomic analysis using the M-IOLITE software suite. IFAC PapersOnLine. (2016) 49:286–8. 10.1016/j.ifacol.2016.12.140

[48] Papadimitropoulos MP Vasilopoulou CG Maga-Nteve C Klapa MI. Untargeted GC-MS metabolomics. Methods Mol Biol. (2018) 1738:133–47. 10.1007/978-1-4939-7643-0_9 29654587

[49] Kanani HH Klapa MI. Data correction strategy for metabolomics analysis using gas chromatography–mass spectrometry. Metab Eng. (2007) 9:39–51. 10.1016/j.ymben.2006.08.001 17052933

[50] Kanani H Chrysanthopoulos PK Klapa MI. Standardizing GC-MS metabolomics. J Chromatogr B Analyt Technol Biomed Life Sci. (2008) 871:191–201. 10.1016/j.jchromb.2008.04.049 18538643

[51] Saeed AI Bhagabati NK Braisted JC Liang W Sharov V Howe EA. TM4 microarray software suite. Methods Enzymol. (2006) 411:134–93. 10.1016/S0076-6879(06)11009-5 16939790

[52] Putnam KT Wilcox M Robertson-Blackmore E Sharkey K Bergink V Munk-Olsen T. Clinical phenotypes of perinatal depression and time of symptom onset: analysis of data from an international consortium. Lancet Psychiatry. (2017) 4:477–85. 10.1016/S2215-0366(17)30136-0 28476427 PMC5836292

[53] Santos H Jr Tan X Salomon R. Heterogeneity in perinatal depression: how far have we come? A systematic review. Arch Womens Ment Health. (2017) 20:11–23. 10.1007/s00737-016-0691-8 27796597 PMC5507213

[54] Wikman A Axfors C Iliadis SI Cox J Fransson E Skalkidou A. Characteristics of women with different perinatal depression trajectories. J Neurosci Res. (2020) 98:1268–82. 10.1002/jnr.24390 30723972

[55] Caldwell RW Rodriguez PC Toque HA Narayanan SP Caldwell RB. Arginase: a multifaceted enzyme important in health and disease. Physiol Rev. (2018) 98:641–65. 10.1152/physrev.00037.2016 29412048 PMC5966718

[56] Cheung KL Lafayette RA. Renal physiology of pregnancy. Adv Chronic Kidney Dis. (2013) 20:209–14. 10.1053/j.ackd.2013.01.012 23928384 PMC4089195

[57] Coppolino G Leonardi G Andreucci M Bolignano D. Oxidative stress and kidney function: a brief update. Curr Pharm Des. (2018) 24:4794–9. 10.2174/1381612825666190112165206 30648504

[58] Cao YF Li J Zhang Z Liu J Sun XY Feng XF. Plasma levels of amino acids related to urea cycle and risk of type 2 diabetes mellitus in chinese adults. Front Endocrinol. (2019) 10:50. 10.3389/fendo.2019.00050 30833930 PMC6387924

[59] Stamler J Elliott P Kesteloot H Nichols R Claeys G Dyer AR. Inverse relation of dietary protein markers with blood pressure. Findings for 10,020 men and women in the INTERSALT Study. INTERSALT Cooperative Research Group. INTERnational study of SALT and blood pressure. Circulation. (1996) 94:1629–34. 10.1161/01.CIR.94.7.1629 8840854

[60] Perlmuter LC Sarda G Casavant V O'hara K Hindes M Knott PT. A review of orthostatic blood pressure regulation and its association with mood and cognition. Clin Auton Res. (2012) 22:99–107. 10.1007/s10286-011-0145-3 21948454

[61] Van Buskirk JJ Kirsch WM Kleyer DL Barkley RM Koch TH. Aminomalonic acid: identification in *Escherichia coli* and atherosclerotic plaque. Proc Natl Acad Sci USA. (1984) 81:722–5. 10.1073/pnas.81.3.722 6366787 PMC344907

[62] Copley SD Frank E Kirsch WM Koch TH. Detection and possible origins of aminomalonic acid in protein hydrolysates. Anal Biochem. (1992) 201:152–7. 10.1016/0003-2697(92)90188-D 1621954

[63] Chen JJ Bai SJ Li WW Zhou CJ Zheng P Fang L. Urinary biomarker panel for diagnosing patients with depression and anxiety disorders. Transl Psychiatry. (2018) 8:192. 10.1038/s41398-018-0245-0 30232320 PMC6145889

[64] Lent-Schochet D Mclaughlin M Ramakrishnan N Jialal I. Exploratory metabolomics of metabolic syndrome: a status report. World J Diabetes. (2019) 10:23–36. 10.4239/wjd.v10.i1.23 30697368 PMC6347655

[65] Rieger E Rech VC Feksa LR Wannmacher CM. Intraperitoneal glycerol induces oxidative stress in rat kidney. Clin Exp Pharmacol Physiol. (2008) 35:928–33. 10.1111/j.1440-1681.2008.04942.x 18430056

[66] Zhang Y Yuan S Pu J Yang L Zhou X Liu L. Integrated metabolomics and proteomics analysis of hippocampus in a rat model of depression. Neuroscience. (2018) 371:207–20. 10.1016/j.neuroscience.2017.12.001 29237567

[67] Boehm G Cervantes H Georgi G Jelinek J Sawatzki G Wermuth B. Effect of increasing dietary threonine intakes on amino acid metabolism of the central nervous system and peripheral tissues in growing rats. Pediatr Res. (1998) 44:900–6. 10.1203/00006450-199812000-00013 9853925

[68] Gall WE Beebe K Lawton KA Adam KP Mitchell MW Nakhle PJ. alpha-hydroxybutyrate is an early biomarker of insulin resistance and glucose intolerance in a nondiabetic population. PLoS ONE. (2010) 5:e10883. 10.1371/journal.pone.0010883 20526369 PMC2878333

[69] Landaas S. The formation of 2-hydroxybutyric acid in experimental animals. Clin Chim Acta. (1975) 58:23–32. 10.1016/0009-8981(75)90481-7 164303

[70] Engin A. The definition and prevalence of obesity and metabolic syndrome. Adv Exp Med Biol. (2017) 960:1–17. 10.1007/978-3-319-48382-5_1 28585193

[71] Hootman KC Trezzi JP Kraemer L Burwell LS Dong X Guertin KA. Erythritol is a pentose-phosphate pathway metabolite and associated with adiposity gain in young adults. Proc Natl Acad Sci USA. (2017) 114:E4233–40. 10.1073/pnas.1620079114 28484010 PMC5448202

[72] Yokozawa T Kim HY Cho EJ. Erythritol attenuates the diabetic oxidative stress through modulating glucose metabolism and lipid peroxidation in streptozotocin-induced diabetic rats. J Agric Food Chem. (2002) 50:5485–9. 10.1021/jf020168z 12207496

[73] Preissler T Bristot IJ Costa BM Fernandes EK Rieger E Bortoluzzi VT. Phenylalanine induces oxidative stress and decreases the viability of rat astrocytes: possible relevance for the pathophysiology of neurodegeneration in phenylketonuria. Metab Brain Dis. (2016) 31:529–37. 10.1007/s11011-015-9763-0 26573865

[74] Camp KM Parisi MA Acosta PB Berry GT Bilder DA Blau N. Phenylketonuria Scientific Review Conference: state of the science and future research needs. Mol Genet Metab. (2014) 112:87–122. 10.1016/j.ymgme.2014.02.013 24667081

[75] Chrousos GP Torpy DJ Gold PW. Interactions between the hypothalamic-pituitary-adrenal axis and the female reproductive system: clinical implications. Ann Intern Med. (1998) 129:229–40. 10.7326/0003-4819-129-3-199808010-00012 9696732

[76] Ang JE Revell V Mann A Mantele S Otway DT Johnston JD. Identification of human plasma metabolites exhibiting time-of-day variation using an untargeted liquid chromatography-mass spectrometry metabolomic approach. Chronobiol Int. (2012) 29:868–81. 10.3109/07420528.2012.699122 22823870 PMC3433180

[77] Davies SK Ang JE Revell VL Holmes B Mann A Robertson FP. Effect of sleep deprivation on the human metabolome. Proc Natl Acad Sci USA. (2014) 111:10761–6. 10.1073/pnas.1402663111 25002497 PMC4115565

[78] Gadad BS Jha MK Czysz A Furman JL Mayes TL Emslie MP. Peripheral biomarkers of major depression and antidepressant treatment response: current knowledge and future outlooks. J Affect Disord. (2018) 233:3–14. 10.1016/j.jad.2017.07.001 28709695 PMC5815949

[79] Meltzer-Brody S. New insights into perinatal depression: pathogenesis and treatment during pregnancy and postpartum. Dialogues Clin Neurosci. (2011) 13:89–100. 10.31887/DCNS.2011.13.1/smbrody 21485749 PMC3181972

[80] Meltzer-Brody S Howard LM Bergink V Vigod S Jones I Munk-Olsen T. Postpartum psychiatric disorders. Nat Rev Dis Primers. (2018) 4:18022. 10.1038/nrdp.2018.22 29695824

[81] Postpartum Depression: Action Towards C Treatment C. Heterogeneity of postpartum depression: a latent class analysis. Lancet Psychiatry. (2015) 2:59–67. 10.1016/S2215-0366(14)00055-8 26359613 PMC4800818

