# Supplementary material for: Metabolic Profiling Indicates Diversity in the Metabolic Physiologies Associated With Maternal Postpartum Depressive Symptoms
Source: Front Psychiatry. 2021 Jun 25;12:685656. doi: 10.3389/fpsyt.2021.685656 (PMC8267859; doi:10.3389/fpsyt.2021.685656)
Supplement: Supplementary file 1 [file Data_Sheet_1.PDF]

Supp. Table 1: The normalized GC-MS metabolomic dataset

Peak Name	Metabolite Name	RT	Quan Ion	CC	Technical CoV%	Aliquot_CoV%	MIN	MEDIAN	MEAN	MAX	C_SAMPLE_1	C_SAMPLE_7	C_SAMPLE_8	C_SAMPLE_11	C_SAMPLE_12	C_SAMPLE_14	C_SAMPLE_15	C_SAMPLE_25	C_SAMPLE_30	C_SAMPLE_31	C_SAMPLE_32	C_SAMPLE_36	PPD_SAMPLE_2	PPD_SAMPLE_9	PPD_SAMPLE_10	PPD_SAMPLE_13	PPD_SAMPLE_16	PPD_SAMPLE_17	PPD_SAMPLE_18	PPD_SAMPLE_19	PPD_SAMPLE_26	PPD_SAMPLE_33	PPD_SAMPLE_34	PPD_SAMPLE_35
2-hydroxybutanoic acid (2TMS)	2-hydroxybutanoic acid	10.072	191+117	1	4.68%	5.08%	0.011	0.070	0.087	0.239	0.064	0.060	0.015	0.074	0.023	0.092	0.076	0.128	0.080	0.041	0.067	0.040	0.164	0.031	0.239	0.034	0.043	0.087	0.157	0.153	0.066	0.011	0.150	0.196
3-methylbenzoate (1TMS)	3-methylbenzoate	18.436	193	1	11.79%	14.64%	0.002	0.005	0.005	0.010	0.005	0.006	0.005	0.005	0.005	0.005	0.004	0.004	0.003	0.004	0.005	0.003	0.005	0.010	0.005	0.004	0.004	0.005	0.004	0.004	0.005	0.002	0.004	0.004
4-hydroxybutanoic acid (2TMS)	4-hydroxybutanoic acid	12.756	233	1	8.56%	20.43%	0.004	0.006	0.006	0.010	0.008	0.010	0.007	0.006	0.005	0.004	0.005	0.004	0.008	0.008	0.005	0.008	0.006	0.006	0.006	0.006	0.006	0.006	0.008	0.007	0.006	0.006	0.005	0.004
aminomalonic acid (3TMS)	aminomalonic acid	19.704	218	1	9.53%	28.64%	0.005	0.018	0.020	0.053	0.013	0.020	0.019	0.016	0.019	0.005	0.007	0.015	0.018	0.025	0.016	0.017	0.020	0.031	0.010	0.010	0.047	0.021	0.025	0.025	0.013	0.027	0.053	0.008
cholesterol (1TMS)	cholesterol	48.586	368	1	4.80%	12.60%	0.176	0.274	0.292	0.617	0.243	0.460	0.338	0.280	0.254	0.259	0.205	0.201	0.199	0.249	0.326	0.291	0.235	0.267	0.355	0.295	0.199	0.617	0.315	0.312	0.176	0.372	0.251	0.306
erythritol (4TMS)	erythritol	17.734	217	1	11.15%	5.88%	0.003	0.005	0.005	0.007	0.005	0.004	0.005	0.006	0.004	0.004	0.003	0.004	0.004	0.004	0.005	0.004	0.005	0.005	0.004	0.005	0.005	0.005	0.007	0.004	0.005	0.005	0.006	0.004
erythronic acid (4TMS)	erythronic acid	19.6	292	1	4.47%	23.98%	0.005	0.053	0.053	0.104	0.055	0.057	0.052	0.052	0.009	0.104	0.036	0.068	0.076	0.033	0.042	0.049	0.022	0.037	0.087	0.074	0.005	0.079	0.053	0.077	0.061	0.052	0.017	0.083
gluconic acid (6TMS)	gluconate	27.995	333	1	5.17%	14.69%	0.005	0.027	0.025	0.039	0.030	0.023	0.021	0.029	0.006	0.035	0.028	0.026	0.033	0.024	0.029	0.029	0.014	0.023	0.034	0.039	0.005	0.026	0.031	0.025	0.021	0.028	0.008	0.037
glucose_total (glucose MeOx1+ glucopyranose 1+ glucopyranose 2)	glucose_total	26.467/26.602/28.286	319/204/204	2	14.76%	6.78%	3.816	5.175	5.199	6.947	4.584	5.285	5.532	4.711	5.379	3.816	5.279	4.696	5.183	5.082	6.112	5.725	4.670	5.619	4.249	5.078	5.167	4.808	6.947	5.476	5.067	6.120	5.447	4.754
glutamate effective (glutamate (3TMS) / pyroglutamate (2TMS))	glutamate	22.628/23.013	246/(230+156)	3	6.89%	16.30%	0.195	0.317	0.329	0.515	0.195	0.434	0.268	0.352	0.226	0.515	0.260	0.425	0.362	0.318	0.316	0.302	0.234	0.312	0.365	0.412	0.237	0.499	0.300	0.438	0.321	0.199	0.222	0.387
glycerate (3TMS)	glycerate	14.922	292	1	6.19%	18.09%	0.006	0.035	0.034	0.058	0.037	0.035	0.030	0.039	0.006	0.058	0.031	0.035	0.047	0.026	0.030	0.035	0.013	0.023	0.055	0.051	missing	0.040	0.032	0.044	0.027	0.030	0.010	0.047
glycerol (3TMS)	glycerol	12.094	205	1	4.92%	5.46%	0.016	0.028	0.031	0.066	0.031	0.018	0.020	0.023	0.023	0.043	0.016	0.020	0.017	0.027	0.040	0.026	0.057	0.026	0.047	0.032	0.028	0.030	0.029	0.037	0.031	0.020	0.066	0.031
glyoxylate (1TMS)	glyoxylate	7.373	160	1	10.83%	21.09%	0.007	0.065	0.068	0.113	0.068	0.058	0.088	0.088	0.019	0.094	0.065	0.090	0.064	0.054	0.060	0.066	0.050	0.056	0.113	0.096	0.007	0.105	0.054	0.058	0.064	0.106	0.026	0.083
isoleucine effective (isoleucine (1TMS) / isoleucine (2TMS))	isoleucine	11.694/13.367	86/158	3	10.42%	14.87%	0.015	0.023	0.022	0.034	0.022	0.026	0.022	0.026	0.028	0.017	0.017	0.015	0.023	0.029	0.016	0.020	0.024	0.031	0.023	0.023	0.034	0.026	0.024	0.021	0.023	0.015	0.021	0.015
lactate (2TMS)	lactate	7.057	117	1	3.42%	6.10%	0.174	0.294	0.311	0.786	0.377	0.235	0.312	0.278	0.267	0.233	0.180	0.270	0.174	0.366	0.445	0.237	0.533	0.313	0.295	0.786	0.202	0.219	0.346	0.312	0.294	0.308	0.187	0.302
leucine effective (leucine (1TMS) / leucine (2TMS))	leucine	11.285/12.706	86/158	3	13.41%	13.97%	0.014	0.022	0.024	0.045	0.018	0.018	0.028	0.024	0.045	0.021	0.014	0.021	0.021	0.028	0.019	0.016	0.024	0.038	0.026	0.033	0.037	0.015	0.022	0.021	0.025	0.018	0.022	0.020
9,12-(Z,Z)-octadecadienoic (linoleic) acid (1TMS)	linoleic acid	34.536	337	1	13.41%	13.80%	0.011	0.016	0.017	0.030	0.015	0.022	0.013	0.017	0.011	0.021	0.012	0.016	0.013	0.015	0.020	0.016	0.020	0.014	0.030	0.016	0.011	0.026	0.017	0.021	0.015	0.015	0.018	0.019
lysine (4TMS)	lysine	27.105	174	3	16.50%	21.64%	0.006	0.014	0.016	0.037	0.007	0.015	0.021	0.018	0.031	0.006	0.008	0.013	0.009	0.018	0.011	0.012	0.017	0.033	0.011	0.010	0.037	0.008	0.017	0.015	0.024	0.011	0.025	0.009
myo-inositol (6TMS)	myo-inositol	29.019	305	1	3.98%	2.63%	0.036	0.042	0.044	0.072	0.047	0.047	0.044	0.046	0.037	0.036	0.036	0.041	0.046	0.038	0.052	0.037	0.041	0.072	0.054	0.046	0.045	0.038	0.040	0.054	0.037	0.036	0.042	0.038
octadecanoic (stearic) acid (1TMS)	octadecanoic acid (stearate)	34.236	117	1	8.87%	15.65%	0.099	0.177	0.184	0.322	0.197	0.235	0.177	0.169	0.135	0.188	0.142	0.161	0.126	0.166	0.184	0.159	0.203	0.185	0.278	0.178	0.099	0.322	0.196	0.214	0.162	0.146	0.156	0.239
ornithine/arginine (4TMS)	ornithine/arginine	25.078	142	3	14.87%	23.22%	0.004	0.009	0.011	0.027	0.006	0.007	0.012	0.021	0.016	0.004	0.005	0.010	0.006	0.012	0.008	0.006	0.012	0.016	0.006	0.007	0.027	0.008	0.015	0.011	0.016	0.006	0.019	0.006
phenylalanine (2TMS)	phenylalanine	23.721	218	3	8.92%	14.65%	0.016	0.023	0.022	0.032	0.019	0.020	0.025	0.027	0.027	0.018	0.017	0.017	0.025	0.023	0.017	0.020	0.021	0.032	0.028	0.025	0.027	0.016	0.022	0.025	0.024	0.019	0.021	0.025
phosphate (4TMS)	phosphate	14.646	387	1	14.42%	17.26%	0.036	0.099	0.092	0.168	0.052	0.092	0.081	0.142	0.111	0.075	0.074	0.082	0.100	0.123	0.116	0.102	0.045	0.100	0.100	0.099	0.168	0.051	0.115	0.136	0.041	0.036	0.107	0.062
serine effective (serine (2TMS) / serine (3TMS))	serine	14.222/15.422	116/204	3	19.38%	29.26%	0.011	0.024	0.026	0.065	0.016	0.022	0.024	0.035	0.031	0.011	0.011	0.021	0.022	0.044	0.020	0.023	0.037	0.036	0.024	0.016	0.065	0.012	0.027	0.024	0.038	0.028	0.032	0.017
sorbitol (6TMS)	sorbitol	26.006	319	1	14.82%	12.03%	0.022	0.033	0.034	0.051	0.036	0.027	0.032	0.045	0.029	0.031	0.044	0.040	0.036	0.034	0.038	0.025	0.024	0.030	0.051	0.030	0.024	0.034	0.030	0.034	0.042	0.028	0.022	0.050
tetradecanoic (myristic) acid (1TMS)	tetradecanoic acid (myristate)	27.132	285	1	5.14%	7.86%	0.010	0.016	0.017	0.027	0.018	0.020	0.012	0.015	0.015	0.018	0.013	0.013	0.012	0.017	0.023	0.014	0.022	0.016	0.027	0.014	0.011	0.020	0.017	0.014	0.017	0.010	0.024	0.017
threonate (4TMS)	threonate	20.39	292+319	1	6.33%	8.50%	0.003	0.013	0.013	0.022	0.019	0.022	0.020	0.018	0.007	0.009	0.013	0.009	0.017	0.013	0.015	0.015	0.006	0.017	0.022	0.016	0.003	0.007	0.007	0.012	0.012	0.013	0.010	0.020
threonine_effective (threonine (2TMS) / threonine (3TMS))	threonine	14.817/15.818	(219+130)/(291+218)	3	9.20%	16.44%	0.010	0.024	0.025	0.045	0.020	0.010	0.024	0.027	0.025	0.022	0.011	0.036	0.019	0.028	0.023	0.015	0.021	0.033	0.032	0.024	0.038	0.024	0.023	0.024	0.045	0.020	0.024	0.027
Un_0017 (P1091/C_041)	Un_0017 (P1091/C_041)	10.834	220	1	12.27%	26.91%	0.057	0.096	0.099	0.182	0.101	0.075	0.073	0.073	0.099	0.101	0.100	0.097	0.099	0.109	0.102	0.088	0.132	0.091	0.107	0.089	0.182	0.182	0.095	0.083	0.068	0.057	0.094	0.086
Un_0063 (a_39/x_2/U_032, RT:20.878/QI:263)	Un_0063 (a_39/x_2/U_032, RT:20.878/QI:263)	21.005	263	1	4.75%	12.76%	0.010	0.015	0.016	0.037	0.020	0.016	0.019	0.012	0.020	0.016	0.014	0.012	0.011	0.015	0.019	0.014	0.014	0.016	0.014	0.016	0.016	0.037	0.016	0.014	0.013	0.016	0.013	0.010
Un_0089 (P1933/f_106/a_33)	Un_0089 (P1933/f_106/a_33)	19.31	232	1	11.79%	25.50%	0.010	0.021	0.023	0.042	0.017	0.017	0.014	0.018	0.019	0.012	0.012	0.028	0.020	0.032	0.024	0.025	0.025	0.033	0.010	0.042	0.021	0.030	0.020	0.035	0.021	0.024	0.022	0.018
Un_0180 (P2555/A_100/U_044, sugar)	Un_0180 (P2555/A_100/U_044, sugar)	25.51	217	1	12.04%	28.01%	0.009	0.014	0.016	0.030	0.010	0.021	0.017	0.012	0.014	0.011	0.014	0.013	0.016	0.014	0.021	0.019	0.011	0.019	0.009	0.012	0.010	0.019	0.030	0.021	0.012	0.027	0.017	0.011
Un_0244 (RT:29.5/QI:218)	Un_0244 (RT:29.5/QI:218)	29.609	218	1	15.90%	28.71%	0.020	0.034	0.038	0.095	0.020	0.037	0.045	0.071	0.031	0.023	0.026	0.026	0.054	0.057	0.030	0.036	0.027	0.095	0.047	0.050	0.028	0.020	0.035	0.048	0.035	0.032	0.023	0.027
Unknown_PPD009 (RT:18.3/QI:200)	unknown_PPD009 (RT:18.3/QI:200)	18.297	200	1	12.33%	28.81%	0.002	0.004	0.004	0.007	0.005	0.007	0.005	0.004	0.003	0.002	0.003	0.002	0.006	0.005	0.004	0.006	0.004	0.004	0.004	0.004	0.004	0.004	0.006	0.005	0.005	0.004	0.003	0.003
Unrev_159 (P2311, pot. dodecanoic (lauric) acid)	Unrev_159 (P2311, pot. dodecanoic (lauric) acid)	23.076	257	1	10.67%	8.22%	0.002	0.005	0.005	0.010	0.007	0.004	0.004	0.004	0.005	0.008	0.004	0.004	0.004	0.006	0.006	0.005	0.009	0.006	0.007	0.004	0.008	0.005	0.004	0.004	0.005	0.002	0.010	0.004
urea (2TMS)	urea	14.962	189	1	8.42%	5.51%	1.450	1.990	1.987	2.651	1.882	2.127	2.084	2.599	2.345	1.729	1.914	1.613	2.020	1.457	1.720	2.054	1.532	1.450	2.160	2.079	1.464	2.376	2.445	1.959	2.399	2.651	1.853	1.780
uric acid (4TMS)	uric acid	34.21	441	1	21.08%	30.11%	0.027	0.239	0.215	0.357	0.206	0.267	0.259	0.245	0.029	0.136	0.142	0.225	0.153	0.297	0.158	0.236	0.201	0.241	0.285	0.357	0.027	0.320	0.325	0.246	0.327	0.180	0.028	0.277
valine effective (valine (1TMS) / valine (2TMS))	valine	9.310/11.056	72/218	3	13.33%	13.49%	0.017	0.033	0.036	0.074	0.037	0.031	0.046	0.037	0.074	0.028	0.025	0.027	0.031	0.041	0.028	0.024	0.035	0.052	0.039	0.048	0.059	0.024	0.030	0.028	0.042	0.017	0.045	0.026

Notes:
1. RT, QI stand for retention time and quantifying ion respectively.
2. Chemical Category is a number 1, 2 or 3 referring to categorization of metabolites based on their derivatization pattern as described in Kanani and Klapa (2007).
3. In some metabolites (mostly unknown), other names already used in publications of the group are provided in parenthesis to enable matching between datasets
4. For metabolites for which an effective relative peak area has been estimated based on the weighted summation of multiple derivatives, the RTs and QIs of these derivatives are shown in the RT and QI cell respectively (separated by /); the names of the derivatives are indicated in the Peak Name cell also separated by /
